# Improving the delivery of health care to patients: radiographers and frontline image interpretation

**DOI:** 10.1002/jmrs.267

**Published:** 2018-03-12

**Authors:** Marilyn Baird

**Affiliations:** ^1^ Foundation Head Department of Medical Imaging and Radiation Sciences School of Primary and Allied Health Care Clayton Victoria Australia; ^2^ Faculty of Medicine, Nursing and Health Sciences Monash University Clayton Victoria Australia; ^3^ Associate Dean Learning and Teaching Faculty of Medicine, Nursing and Health Sciences Monash University Clayton Victoria Australia

## Abstract

Australia led the world in raising the level of entry‐level education to practice as a radiographer. It now lags behind in formalising radiographer input into the process of image interpretation. The time has come to rectify this situation.

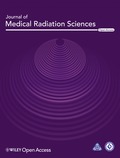

In September 2016, I attended the inaugural ‘International Radiographer Advanced Practice Conference’ hosted by Sheffield Hallam University with support from local and international professional bodies. The conference theme ‘Leading the Way’ was apposite given the part played by the host and The Society of Radiographers in advancing radiography practice to the effect that United Kingdom (UK) registered radiographers are now expected to provide a ‘Preliminary Clinical Evaluation’ or PCE,^1^ which involves knowing how to detect an abnormality and how to follow‐up with a written description.^2^

While during the conference, there was a spirited debate as to the level of academic achievement that the clinical consultant radiographers should achieve, at least the UK has a clear career progression pathway for radiographers requiring post‐graduate degree to achieve the status of reporting radiographer.^1^ Australian representatives lamented the lack of genuine progress along the continuum from entry level to advanced practitioner despite the fact that we had led the way in introducing degree level education in 1985 and treat computed tomography (CT) as an entry level ‘competency’. Certainly, professional advancement happens in Australia but arguably it is based on expertise in an imaging modality. Therefore, many move away from general radiography as it is seen to be lacking technological challenge and formal input into diagnosis.

The Australian literature around radiographer advancement confirms radiographers continue to face multiple road blocks on the pathway to achieve recognition for a role in image interpretation let alone advanced practitioner status^3^, ^4^, ^5^, ^6^ The reaction by the Royal Australian and New Zealand College of Radiologists (RANZCR) to the 2007 suggestion from Smith and Baird^7^ that within a framework of ‘task substitution’ radiographers could play a role in relieving radiologists of the burden associated with reporting images of the appendicular skeleton, reflected the traditional argument that medical knowledge is essential to interpreting images.^8^ In 2018, one has to ask if RANZCR objections to any form of formalised image interpretation on the part of radiographers still hold true. Notwithstanding the fact that the UK achievements may only ever be fully realised within a national health system, I am of the view that Australian state‐based health authorities can be confident that appropriately educated radiographers are able to provide support at the coal face where we know junior doctors can struggle to accurately interpret trauma radiographic images^9^, ^10^, ^11^, ^12^ This is not to say that ‘red dot’ detection systems and radiography commenting processes are currently absent from our workplaces^3^, ^4^ What is missing is the wholesale adoption of a formal process whereby radiographers are obliged to engage in a detection and recording process. What then has changed since 2007?

First, radiographers in Australia are now registered within the National Registration and Accreditation Scheme and need to align their practice with the expectations of the Medical Radiation Practice Board of Australia (MRPBA). Next, the Board has chosen to describe its minimum expectations about entry level ‘knowledge’, ‘skills’ and ‘professional attributes’ through a series of ‘Capability Statements’,^13^ which attest to the genuine complexity associated with the delivery of a clinically grounded professional practice. These statements provide the flexibility to respond to new challenges in health care delivery, which is certainly the case in Australia with respect to the current collaborative care, indigenous health and safety and quality imperatives.^14^ Domain 5 of the statements, in particular, confirms (the fact) that radiographers are part of multiprofessional health care teams with responsibilities to collaborate. While it came as a surprise to some radiographers that the expectation to ‘convey information [to appropriate members of the health care team] when significant findings are identified’^14^ is standard practice,^15^ the obligation to pass on useful insights regarding image findings is now obligatory. From the Board's perspective, ‘identifying significant findings includes recognising and applying knowledge of normal from abnormal imaging appearances and relating appearances to the patient/client's clinical history’^14^ including CT. Furthermore, the Accreditation Committee of the MRPBA^16^ lists the clinical conditions necessitating an opinion from radiographers in its advice to universities seeking to apply for accreditation of their medical imaging courses.^16^

Second, undergraduate radiography degrees have by and large moved from three to four years at either Australian Qualification Level 7 or 8.^17^ This change is not only wholly related to ensuring students achieve appropriate levels of clinical expertise but also ensures students are educated in relation to a broad range of medical imaging modalities including image interpretation, psychophysics of vision and gross pathology. Many universities, including my own, also offer online Masters programs in image interpretation. Finally, the papers I have cited confirm that radiographer practitioners and academics have the requisite intellectual training to engage in credible research into role advancement, in particular, in relation to image interpretation.

This year the MRPBA is planning to review its Capability Statements. Now is the time to argue for the requirement that all registered radiographers issue a PCE. While this is not advanced practice, the production of it will require access to ongoing educational support as Neep et al. argue in their paper within this edition of the journal.^18^ The systematic review into educational methods used to train radiographers to interpret chest images, as conducted by McLaughlin et al.,^19^ confirms the necessity for post‐graduate training in relation to achieving accuracy levels that equate with the gold standard which is the radiology report. Therefore, we need to make sure that all image interpretation courses are appropriately constructed with measurable learning outcomes and supported by evidence‐based learning modules, self‐directed learning activities, and a rigorous and defensible assessment regime. Also, completion of a training course whether one provided in‐house or as part of a University accredited Masters’ program must be accompanied by an ongoing audit process and benchmarking within a strong clinical governance framework.^1^, ^11^, ^12^, ^18^, ^19^, ^20^

Where to now? While a degree of success in advanced practice has been achieved in radiation therapy, the same cannot be said for general radiography. Has the time come for those universities currently providing Masters level programs in image interpretation to collaborate and create a Professional Certificate in Musculo–Skeletal Image Interpretation hosted by the Australian Society of Medical Imaging and Radiation Therapy with support from the State‐based Health Departments? I was encouraged during the 3rd Singapore Health Duke‐National University of Singapore Education conference held on 29 and 30 September 2017.^21^ In a session focussed on patient safety radiology, it was confirmed that all radiographers at two of the three health networks engage in a ‘radiograph abnormality detection scheme’ that has unsurprisingly also led to improved standards in the quality of the radiographic images. At the conference, I argued patient safety demands radiographers assume a genuine stake in the diagnostic process otherwise the health sector is losing a vital opinion that has been informed by direct clinical contact with the patient. We cannot wait any longer; it is time to act.

## References

[1] https://www.sor.org/learning/document-library/preliminary-clinical-evaluation-and-clinical-reporting-radiographers-policy-and-practice-guidance (accessed January 6 2018)

[2] Paterson A , Piper KJ . Initial image interpretation of appendicular skeletal radiographs: a comparison between nurses and radiographers. Radiography 2009; 15: 40–8.

[3] Neep MJ , Steffens T , Owen R , McPhail SM . Radiographer commenting of trauma radiographs: a survey of the benefits, barriers and enablers to participation in an Australian healthcare setting. J Med Imaging Radiat Oncol 2014; 58: 431–8. 24774619 10.1111/1754-9485.12181

[4] Neep MJ , Steffens T , Owen R , McPhail SM . A survey of radiographers’ confidence and self‐perceived accuracy in frontline image interpretation and their continuing educational preferences. J Med Radiat Sci 2014; 61: 69–77. 26229640 10.1002/jmrs.48 PMC4175834

[5] Neep MJ , Steffens T , Riley V , Eastgate P , McPhail SM . Development of a valid and reliable test to assess trauma radiograph interpretation performance. Radiography 2017; 23: 153–8. 28390548 10.1016/j.radi.2017.01.004

[6] Squibb K , Bull RM , Smith A , Dalton L . Australian rural radiographers’ perspectives on disclosure of their radiographic opinion to patients. Radiography 2015; 21: 25–9.

[7] Smith TN , Baird M . Radiographers’ role in radiological reporting: a model to support future demand. Med J Aust 2007; 186: 629–31. 17576178 10.5694/j.1326-5377.2007.tb01080.x

[8] Kenny LM , Andrews MW . Addressing radiology workforce issues. Med J Aust 2007; 186: 615–6. 17576174 10.5694/j.1326-5377.2007.tb01076.x

[9] McConnell JR , Baird MA . Could musculo‐skeletal radiograph interpretation by radiographers be a source of support to Australian medical interns: a quantitative evaluation. Radiography 2017; 23: 321–9. 28965896 10.1016/j.radi.2017.07.001

[10] Kelly BS , Rainford LA , Gray J , McEntee MF . Collaboration between radiological technologists (radiographers) and junior doctors during image interpretation improves the accuracy of diagnostic decisions. Radiography 2011; 18: 90–5.

[11] McConnell J , Devaney C , Gordon M . Queensland radiographer clinical descriptions of adult appendicular musculo‐skeletal trauma following a condensed education programme. Radiography 2013; 19: 48–55.

[12] McConnell J , Devaney C , Gordon M , Goodwin M , Strahan R , Baird M . The impact of a pilot education programme on Queensland radiographer abnormality description of adult appendicular musculo‐skeletal trauma. Radiography 2012; 18: 184–90.

[13] https://www.coaghealthcouncil.gov.au/ (accessed 6 January 2018)

[14] http://www.medicalradiationpracticeboard.gov.au/Registration/Professional-Capabilities.aspx (accessed 6 January 2018)

[15] http://www.medicalradiationpracticeboard.gov.au/News/Newsletters/October-2017.aspx (accessed 6 January 2018)

[16] http://www.medicalradiationpracticeboard.gov.au/Accreditation/Application-information.aspx (accessed 6 January 2018)

[17] http://www.medicalradiationpracticeboard.gov.au/Accreditation.aspx (accessed 6 January 2018)

[18] Neep M , Steffens T , Eastgate P , McPhail S . Evaluating the effectiveness of intensive versus non‐intensive image interpretation education for radiographers: a randomised control trial study protocol. J Med Radiat Sci 2018; 65: 63–70. 10.1002/jmrs.264 PMC5846025 29388344

[19] McLaughlin L , McConnell J , McFadden S , Bond R , Hughes C . Methods employed for chest radiograph interpretation education for radiographers: a systematic review of the literature. Radiography 2017; 23: 350–7. 28965900 10.1016/j.radi.2017.07.013

[20] Wright C , Reeves P . RadBench: benchmarking image interpretation skills. Radiography 2016; 22: e131–6.

[21] https://www.academic-medicine.edu.sg/educationconference2017/ (accessed 7 January 2018)

